# Comparative Study of a Real-Time PCR Assay Targeting senX3-regX3 versus Other Molecular Strategies Commonly Used in the Diagnosis of Tuberculosis

**DOI:** 10.1371/journal.pone.0143025

**Published:** 2015-11-24

**Authors:** Rocio Sanjuan-Jimenez, Inmaculada Toro-Peinado, Pilar Bermudez, Juan D. Colmenero, Pilar Morata

**Affiliations:** 1 Biochemistry, Molecular Biology and Immunology Department, Faculty of Medicine, University of Malaga, Malaga, Spain; 2 Microbiology Service, Regional University Hospital, Malaga, Spain; 3 Infectious Diseases Service, Regional University Hospital, Malaga, Spain; National Environmental Engineering Research Institute CSIR, INDIA

## Abstract

**Background:**

Nucleic acid amplification tests are increasingly used for the rapid diagnosis of tuberculosis. We undertook a comparative study of the efficiency and diagnostic yield of a real-time PCR senX3-regX3 based assay versus the classical IS6110 target and the new commercial methods.

**Methods:**

This single-blind prospective comparative study included 145 consecutive samples: 76 from patients with culture-confirmed tuberculosis (86.8% pulmonary and 13.2% extrapulmonary tuberculosis: 48.7% smear-positive and 51.3% smear-negative) and 69 control samples (24 from patients diagnosed with non-tuberculous mycobacteria infections and 45 from patients with suspected tuberculosis which was eventually ruled out). All samples were tested by two CE-marked assays (Xpert^®^MTB/RIF and AnyplexTM plus MTB/NTM) and two in-house assays targeting senX3-regX3 and the IS6110 gene.

**Results:**

The detection limit ranged from 1.00E+01 fg for Anyplex, senX3-regX3 and IS6110 to 1.00E+04 fg for Xpert. All three Xpert, senX3-regX3 and IS6110 assays detected all 37 smear-positive cases. Conversely, Anyplex was positive in 34 (91.9%) smear-positive cases. In patients with smear-negative tuberculosis, differences were observed between the assays; Xpert detected 22 (56.41%) of the 39 smear-negative samples, Anyplex 24 (61.53%), senX3-regX3 28 (71.79%) and IS6110 35 (89.74%). Xpert and senX3-regX3 were negative in all control samples; however, the false positive rate was 8.7% and 13% for Anyplex and IS6110, respectively. The overall sensitivity was 77.6%, 85.7%, 77.3% and 94.7% and the specificity was 100%, 100%, 90.8% and 87.0% for the Xpert, senX3-regX3, Anyplex and IS6110 assays, respectively.

**Conclusion:**

Real-time PCR assays targeting IS6110 lack the desired specificity. The Xpert MTB/RIF and in-house senX3-regX3 assays are both sensitive and specific for the detection of MTBC in both pulmonary and extrapulmonary samples. Therefore, the real time PCR senX3-regX3 based assay could be a useful and complementary tool in the diagnosis of tuberculosis.

## Introduction

Tuberculosis (TB) remains a major public health problem in many underdeveloped and developing countries. The 9 million new cases and 1.5 million deaths directly attributable to TB in 2013 are a clear expression of the importance of this infection [1]. Accurate and rapid diagnosis of pulmonary TB, including smear-negative cases, is the cornerstone for TB control. In addition, the morbidity and mortality of both pulmonary and extrapulmonary TB are directly related to diagnostic delay. However, as the microscopic examination lacks adequate sensitivity and culturing is labour intensive and time consuming [2–4], interest is increasing in molecular methods for the diagnosis of TB. Among these latter, real-time PCR has been shown to be the most cost-effective in clinical laboratories [5–6].

Although culture is still considered the gold standard and is the first step in detecting drug resistance, molecular techniques can be automated and are rapid, which explains why they are gaining importance in the diagnosis of TB. Until very recently, IS6110 has been the target most commonly used for the molecular diagnosis of TB [7–8]. The presence of multiple copies of IS6110 in the genome of most species of *Mycobacterium tuberculosis* complex (MTBC) would increase the sensitivity of any technique based on this insertion sequence [9–12].

Recently, the WHO and the FDA have endorsed Xpert^®^MTB/RIF (GX), a novel, rapid, automated, cartridge-based nucleic acid amplification test that can simultaneously detect TB and rifampicin resistance as a suitable test for the diagnosis of pulmonary and extrapulmonary TB, even in low income countries [13–14]. Another molecular technique, Anyplex™ plus MTB/NTM Detection kit (Anyplex), has also been CE-marked for the simultaneous differential diagnosis of tuberculosis and non-tuberculous mycobacteria (NTM) infection [15]. However, the diagnostic yield of Anyplex is not well established and no studies have yet compared the efficiency of these two commercial assays.

We have previously reported that PCR targeting the senX3-regX3 intergenic region in an in-house multiplex real-time PCR format has proven to be a suitable technique for the diagnosis of extrapulmonary tuberculosis [16–17]. Our aim in the present study was to undertake a comparative analysis of the efficiency and diagnostic yield of our real-time PCR senX3-regX3 based assay versus the classical IS6110 target and other new commercial methods in a representative sample of patients with TB.

## Materials and Methods

### Study design, population and clinical samples

From June 2013 to December 2014 we undertook a single-blind prospective study at Malaga Regional Hospital, a 1100-bed tertiary university hospital located in the south of Spain and covering a population of about 350,000 inhabitants with a medium-to-low prevalence of TB.

**Fig 1:**
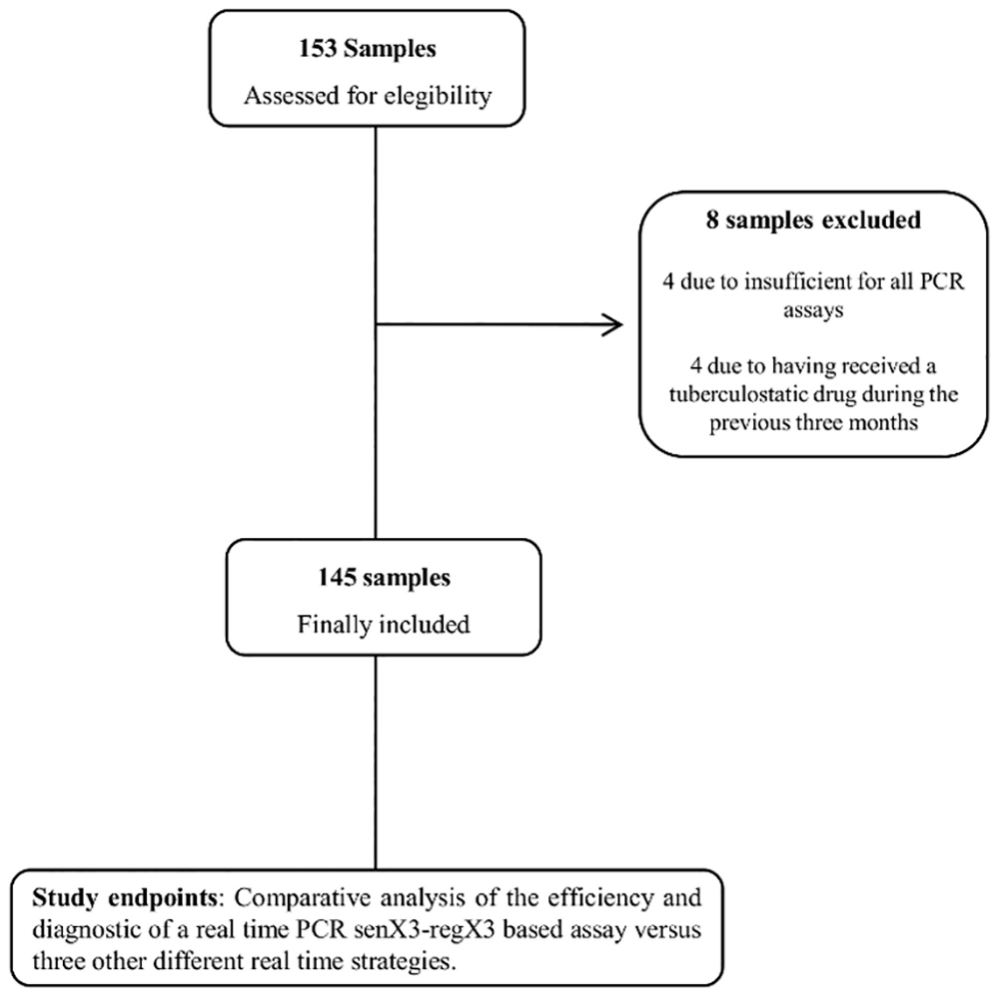
Inclusion process for sample selection.

One hundred and fifty-three samples were initially assessed for eligibility. Eight of them were eventually discarded; 4 due to insufficient sample volume for all PCR assays and 4 due to having received tuberculostatic drugs during the previous three months (Fig 1). Finally, the study included 145 samples from 145 patients aged 2–87 years; 76 samples were from patients diagnosed with culture-confirmed tuberculosis and the remaining 69 were controls. The control group consisted of 24 samples from patients diagnosed with different NTM infections and 45 taken randomly from patients initially suspected to have tuberculosis but which was finally ruled out. Of the 145 samples, 125 were of pulmonary and 20 extrapulmonary origin.

### Bacterial strains and sample processing

The bacterial strains used in this study were provided by the American Types Culture Collection (*M*. *tuberculosis* H37Rv ATCC 27294 and *M*. *bovis* ATCC 19210) and the clinical isolates (*M*. *bovis* and *E*. *coli*) were previously characterized in the microbiology laboratory of the Regional University Hospital of Malaga. MTBC strains were cultured on Lowenstein-Jensen medium (Biomedics) and incubated at 37°C for 2–4 weeks in order to obtain sufficient bacterial growth for later extraction of genomic DNA. The strain of *E*. *coli* was cultured on blood agar at room temperature for 24 h and included as a control. All procedures were performed in a biosafety cabinet class II B3.

The pulmonary (sputum, bronchial aspirate (BAS), bronchoalveolar lavage (BAL) and gastric aspirate), urine and stool samples were decontaminated, homogenized and concentrated by the N-acetyl-L-cysteine-NaOH procedure, using the BBL^®^ Mycoprep kit (Becton Dickinson). Extrapulmonary specimens from sterile sites were not decontaminated and were used directly, while homogenized samples from the different tissues were resuspended in 1 ml of molecular biology water and centrifuged at 15.000x g for 10 min. The supernatant was discarded. These samples were examined by microscopy and culture. Fixed smears were stained with auramine fluorochrome. The smear-positive slides were confirmed by Ziehl-Neelsen staining. All the samples were inoculated on a BACTEC MGIT 960 system (Becton Dickinson Diagnostic Instrument Systems, Sparks, MD) and Lowenstein—Jensen media and were incubated for 6–8 weeks before being discarded. Each sample was also used for Xpert MTB/RIF and DNA extraction. After preliminary identification in the microbiology laboratory of the Regional University Hospital of Malaga, all the isolated strains of MTBC and NTM were later characterized at the Mycobacterium reference laboratory, at the Instituto de Salud Carlos III, Madrid, Spain.

### DNA extraction

DNA from 350 μl of the reference strains and clinical samples was extracted using the QIAamp DNA Mini kit (Qiagen) according to the manufacturer’s instructions. Previously, both samples and strains were heated for 20 min at 80°C and were slightly cooled at room temperature before incubating with 0.05 mg lysozyme for at least 1h at 37°C. To monitor for contamination, negative controls with *E*. *Coli* DNA were included during each DNA extraction procedure. Purified DNA was resuspended in 50 μl of buffer TE 1X and quantified by reading the absorbance at 260 and 280 nm with a ND-1000 spectrophotometer (Nanodrop ThermoFisher).

### Xpert^®^MTB/RIF (Cepheid)

The Xpert MTB/RIF assay was performed using the GeneXpert^®^ instrument (Cepheid), according to the manufacturer’s protocol. Briefly, 0.5–1 ml of each sample was resuspended in 2 ml of the sample reagent and incubated for 20 min at room temperature. The samples were mixed gently with a vortex twice during incubation and then transferred into the Xpert^®^ cartridge. Each clinical sample was tested only once. This hemi-nested real time PCR uses molecular beacon and primers targeting the rpoB gene.

### Anyplex™ plus MTB/NTM detection v1.1 (Seegene)

The multiplex Anyplex™ plus MTB/NTM Detection system was performed following the manufacturer’s instructions on a CFX96™ Real-Time PCR system (Bio-Rad) and Seegene Viewer 2.0 software (Seegene). The multiplex amplification mixture included 1X MTB/NTMplus OM, 1X Anyplex PCR Master Mix, 5μl of template DNA and nuclease-free dH2O adjusted to a final volume of 20 μl. Each clinical sample was tested once, except for invalid results which were repeated. A positive control with MTBC DNA, extraction control with *E*. *coli* DNA and negative control with nuclease-free dH2O were included in each run. The reactions were cycled 45 times, after an initial hold at 50°C for 5 min and 95°C for 15 min, between 95°C for 30 s and 60°C for 1 min with detection of fluorescence on the FAM (MTBC), HEX (genus *Mycobacterium*) and Quasar 670 (internal control) channel. This kit uses Dual Priming Oligonucleotide (DPO) and Real Amplicon Detection (READ) technologies in a multiplex assay with the target 16S rRNA for detection of the *Mycobacterium* genus and the targets IS6110 and mpb64 gene for MTBC.

### IS6110 and senX3-regX3 in-house assays

Two independent assays were evaluated with the IS6110 and senX3-regX3 target. Both real-time PCR assays were performed using a LightCycler^®^ 480 Instrument (LC 480) (Roche), with the LightCycler^®^ 480 SYBR Green I Master kit (Roche), and using the primers IS6110f/IS6110r and M1f/M3r, previously described [18]. The 20 μl amplification mixture included 5 μl of template DNA, 1X master mix, 0.5 μM each forward and reverse primer and nuclease-free dH2O. Each run included a positive control, extraction control and negative control. Before the amplification, the template was initially denatured by incubation at 95°C for 5 min then the cycling conditions were of 95°C for 10 s, 64°C (senX3-regX3) or 58°C (IS6110) for 10 s, and 72°C for 6 or 4 s respectively, with fluorescence acquisition. After cycle 45, melting curves were acquired on the SYBR channel by heating at 95°C for 5 s, cooling at 65°C for 1 min and heating at 5°C/s until 97°C. The Ct values were determined by the second derivative maximum method. Each clinical sample was tested twice and was considered as positive if both replicates showed amplification and appropriate Tm. The first 25 samples of the study were processed in a LightCycler^®^ 2.0 instrument (LC 2.0) (Roche) using the LightCycler^®^ FastStart DNA MasterPlus SYBR Green I kit (Roche) under the same conditions defined above, except for an initial hold at 95°C for 10 min, annealing at 60°C for both targets and monitoring of fluorescence continuously at a ramping rate of 0.1°C/s until 95°C according to the manufacturer’s protocol. Universal precautions and a laminar flow hood were used to prevent contamination.

### Standard curves

DNA from *M*. *tuberculosis* H37Rv ATCC 27294 (4.41 Mb) and *M*. *bovis* ATCC 19210 (4.34 Mb) were used to determine the analytical sensitivity of the assays by testing serial decimal dilutions of genomic DNA in triplicate ranging from 1.00E+08 (approximately 2.00E+07 GE) to 1.00E+01 fg (approximately 2 GE). The number of genome equivalents (GE) was calculated according to the following equation: NGE = Avogadro constant (GE g-1 mol-1) * DNA concentration (C) (ng μl-1)/ Genome bp * molecular mass of 1 bp (g mol-1 bp-1) * 1.00E+09 (ng). Samples in triplicates from a bacterial suspension with OD600 = 0.5 were collected and counted separately using an improved Neubauer counting chamber (C-Chip DHC-N01, Incyto) under an Olympus CX41 microscope. In order to minimize the number of bacterial clumps, all the suspensions were vortexed at full speed for 10 min. Once the total number of *M*. *bovis* was defined, serial decimal dilutions from 1.00E+08 (2.13E+07 GE) to 1.00E+01 fg/ml (2.13 GE) were adjusted.

### Statistical analysis

Continuous variables are represented as mean ± standard deviation and qualitative variables as percentages. The correlation between the Ct values and bacterial inoculum was assessed by log-log regression. Covariance analysis was used to compare Ct values of the senX3-regX3 and IS6110 targets depending on the LC technology used. To assess the diagnostic yield, sensitivity, specificity, positive and negative predictive values, likelihood ratios, accuracy and 95% confidence intervals (CI) were calculated. All tests were two-tailed, and a *p* value <0.05 was considered statistically significant. Data and Figs were performed using GraphPad Prism 6 software.

### Ethics statement

All participants were informed about the objectives and design of the study and a written informed consent form was signed before their inclusion in the study. If the subjects were minors, the written informed consent was given by the parents or legal guardians as appropriate in each case. The Ethics Committees of the Regional University Hospital of Malaga approved the consent procedure and the use of samples for research.

## Results

### Culture and smear results of clinical samples

Of the 76 patients diagnosed with TB, 66 (86.8%) had pulmonary TB and 10 (13.2%) extrapulmonary TB. Of these extrapulmonary cases, the TB was osteoarticular in 4 patients (40%), pleural in 3 patients (30%) and the remaining 3 patients (30%) were one each of lymph node tuberculosis, tuberculous meningitis and renal tuberculosis. Of the 76 patients, 37 (48.7%) had smear-positive samples and 39 (51.3%) smear-negative samples.

Of the 24 patients with NTM infections, 6 were produced by *M*. *avium*, 5 *M*. *intracellulare*, 2 *M*. *mucogenicum*, 2 *M*. *gordonae*, 2 *M*. *lentiflavum*, and one each of *M*. *genavense*, *M*. *chelonae*, *M*. *kansasii*, *M*. *scrofulaceum*, *M*. *novocastrese*, *M*. *fortuitum* and *M*. *abscessus*. Culture and smear microscopy results according to the sample type are shown in Table 1.

**Table 1:** Culture and smear microscopy results according to sample type.

Sample (number)	Culture		Smear	
	+	-	+	-
Sputum (96)	53	43	33	63
BAS (19)	9	10	3	16
BAL (4)	2	2	1	3
Gastric aspirate (6)	2	4	1	5
Pleural fluid (7)	3	4	0	7
Osteoarticular (4)^1^	4	0	1	3
CSF (2)	1	1	0	2
Lymph node (2)	1	1	1	1
Stool (2)	0	2	1	1
Urine (1)	1	0	0	1
Peritoneal fluid (1)	0	1	0	1
Soft tissue (1)^2^	0	1	0	1
T = 145	76	69	41	104

^**1**^Two paravertebral abscess, one vertebral biopsy and sacroiliac joint biopsy.

^2^ Parasternal inflammatory mass.

### Analytical sensitivity

**Fig 2:**
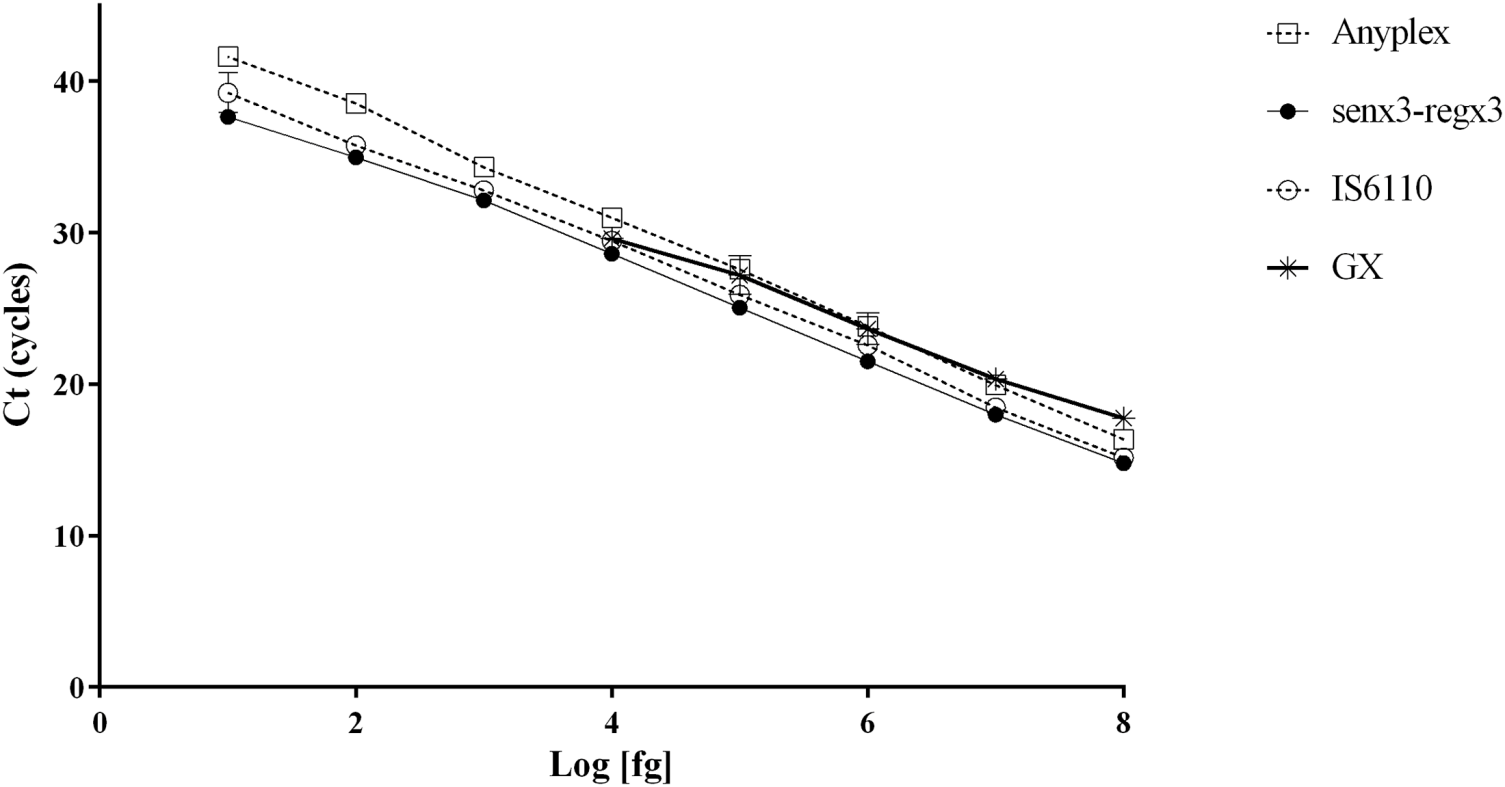
Standard curves of different assays.

Standard curves for the GX, Anyplex, senX3-regX3 and IS6110 assays are shown in Fig 2. The linear regression equations obtained were Ct = -3.057 log C + 42.05 (R2 = 0.996) for GX, Ct = -3.627 log C + 45.37 (R2 = 0.999) for Anyplex, Ct LC 480 = -3.338 log C + 41.61 (R2 = 0.998) for senX3-regX3 and Ct LC 480 = -3.44 log C + 42.92 (R2 = 0.998) for IS6110, with an efficiency of 2.12, 1.88, 1.99 and 1.95 respectively. The limit of detection (LOD) was 1.00E+04 fg (approximately 2.00E+03 GE) for GX and 1.00E+01 fg (approximately 2 GE) for the other three assays. As the senX3-regX3 and IS6110 assays were performed on two different instruments during the study, in order to assess the possible impact on the efficiency of the two assays depending on the instrument used, the results were compared in an intra-assay analysis (S1 Fig).

The DNA concentration range in 10 fold serial dilution tested in triplicate ranged from 1.00E+07 fg (approximately 2.00E+06 GE) to 1.00E+01 fg (approximately 2.00 GE). For senX3-regX3, the linear regressions were Ct LC 2.0 = -3.330 log C + 41.53 (R2 = 0.997) and Ct LC 480 = -3.320 log C + 41.56 (R2 = 0.997); the differences between the slopes were not significant (F = 0.008; P = 0.930). Conversely, for IS6110, the equations were Ct LC 2.0 = -3.991 log C + 44.06 (R2 = 0.997) and Ct LC 480 = -3.441 log C + 42.83 (R2 = 0.998), the differences between the slopes being significant (F = 27.09; P = 0.000). The IS6110 assay on the LC480 instrument was more efficient than on the LC 2.0. Differences between the Tm were also observed for the different instruments and mastermix used; 1.24 ±0.23°C for senX3-regX3 and 0.83 ±0.20°C for IS6110 (S2 Fig).

### Diagnostic yield

The GX, senX3-regX3 and IS6110 assays correctly detected all 37 smear-positive cases, whereas Anyplex was positive in 34 (91.9%) smear-positive cases. In patients with smear-negative TB there were major differences between the different assays. GX detected 22 (56.41%) of the 39 smear-negative samples, Anyplex 24 (61.53%), senX3-regX328 (71.79%) and IS6110 35 (89.74%).

All samples from the control group were negative with the GX and senX3-regX3 assays, whereas the specificity of the Anyplex and IS6110 assays was 90.8% and 87.0%, respectively. False positive results with Anyplex occurred in 4 patients without evidence of infection and 2 NTM (1 *M*. *abscessus* and 1 *M*. *intracellulare*) and with IS6110 in 2 patients without evidence of infection and 7 NTM (3 *M*. *avium*, 1 *M*. *lentiflavum*, 1 *M*. *abscessus*, 1 *M*. *intracellulare*, 1 *M*. *fortuitum*).

Table 2 summarizes the overall diagnostic yield of the four real-time PCR assays analysed. The sensitivity was highest with IS6110, followed by senX3-regX3, Anyplex and GX, but only senX3-regX3 and GX were completely specific. Thus, the overall accuracy was highest for senX3-regX3, followed by GX, IS6110 and Anyplex.

**Table 2:** Overall diagnostic yield of the GX, Anyplex, senX3-regX3 and IS6110 assays.

%, (95% CI)	GX	Anyplex	senX3-regX3	IS6110
**Sensitivity**	77.6, (68.3–87.0)	77.3, (67.9–86.8)	85.5, (77.6–93.4)	94.7, (89.7–99.8)
**Specificity**	100	90.8, (83.7–97.8)	100	87.0, (79.0–94.9)
**PPV**	100	90.6, (83.5–97.8)	100	88.9, (82.0–95.7)
**NPV**	80.2, (71.8–88.6)	77.6, (68.3–87.0)	86.3, (78.7–93.8)	93.8, (87.8–99.7)
**Accuracy**	88.3, (83.0–93.5)	83.6, (77.4–89.7)	92.4, (88.1–96.7)	91.0, (86.4–95.7)
**Positive LR**	NA*	8.4, (3.87–18.13)	NA*	7.3, (3.94–13.39)
**Negative LR**	0.22, (0.15–0.34)	0.25, (0.16–0.38)	0.14, (0.08–0.25)	0.06, (0.02–0.16)

PPV, positive predictive values;

NPV, negative predictive;

Positive LR, positive likelihood ratio;

Negative LR, negative likelihood ratio;

95% CI = 95% confidence interval;

NA*, not assessable.

The partial sensitivity and specificity of the samples categorized as pulmonary and extrapulmonary and according to the smear results are shown in Table 3. In all the assays the sensitivity decreased in smear-negative samples, being more pronounced in pulmonary samples for GX and Anyplex and in extrapulmonary samples for the senX3-regX3 assay. The partial specificity for Anyplex and IS6110 was very similar in smear-negative samples.

**Table 3:** Sensitivity and specificity of the different assays according to the site of infection and smear status.

**Type of sample (smear)**	**Assay**	**TP** ^**^**^	**FP** ^**^**^	**TN** ^**^**^	**FN** ^**^**^	**I** ^**^**^	**Sensitivity** *	**Specificity** *
Pulmonary (smear +)	GX	36	0	2	0	0	100	100
	Anyplex	33	0	2	2	1	94.3 (86.6–102.0)	100
	senX3-regX3	36	0	2	0	0	100	100
	IS6110	36	1	1	0	0	100	50(19.3–119.3)
Pulmonary (smear -)	GX	16	0	57	14	0	53.3 (35.5–71.2)	100
	Anyplex	18	6	48	12	3	60.0 (42.5–77.5)	88.9 (80.5–97.3)
	senX3-regX3	23	0	57	7	0	76.7 (61.5–91.8)	100
	IS6110	27	7	50	3	0	90.0 (79.3–100.7)	87.7 (79.2–96.2)
**Type of sample (smear)**	**Assay**	**TP** ^**^**^	**FP** ^**^**^	**TN** ^**^**^	**FN** ^**^**^	**I** ^**^**^	**Sensitivity** *	**Specificity** *
Extrapulmonary (smear+)	GX	1	0	2	0	0	100	100
	Anyplex	1	0	2	0	0	100	100
	senX3-regX3	1	0	2	0	0	100	100
	IS6110	1	1	1	0	0	100	50 (19.3–119.3)
Extrapulmonary (smear -)	GX	6	0	8	3	0	66.7 (35.9–97.5)	100
	Anyplex	6	0	7	3	1	66.7 (35.9–97.5)	100
	senX3-regX3	5	0	8	4	0	55.6 (23.1–88.0)	100
	IS6110	8	0	8	1	0	88.9 (68.4–109.4)	100

*95% CI

^ TP, true positive;

FP, false positive;

TN, true negative;

FN, false negative;

I, invalid result.

**Fig 3:**
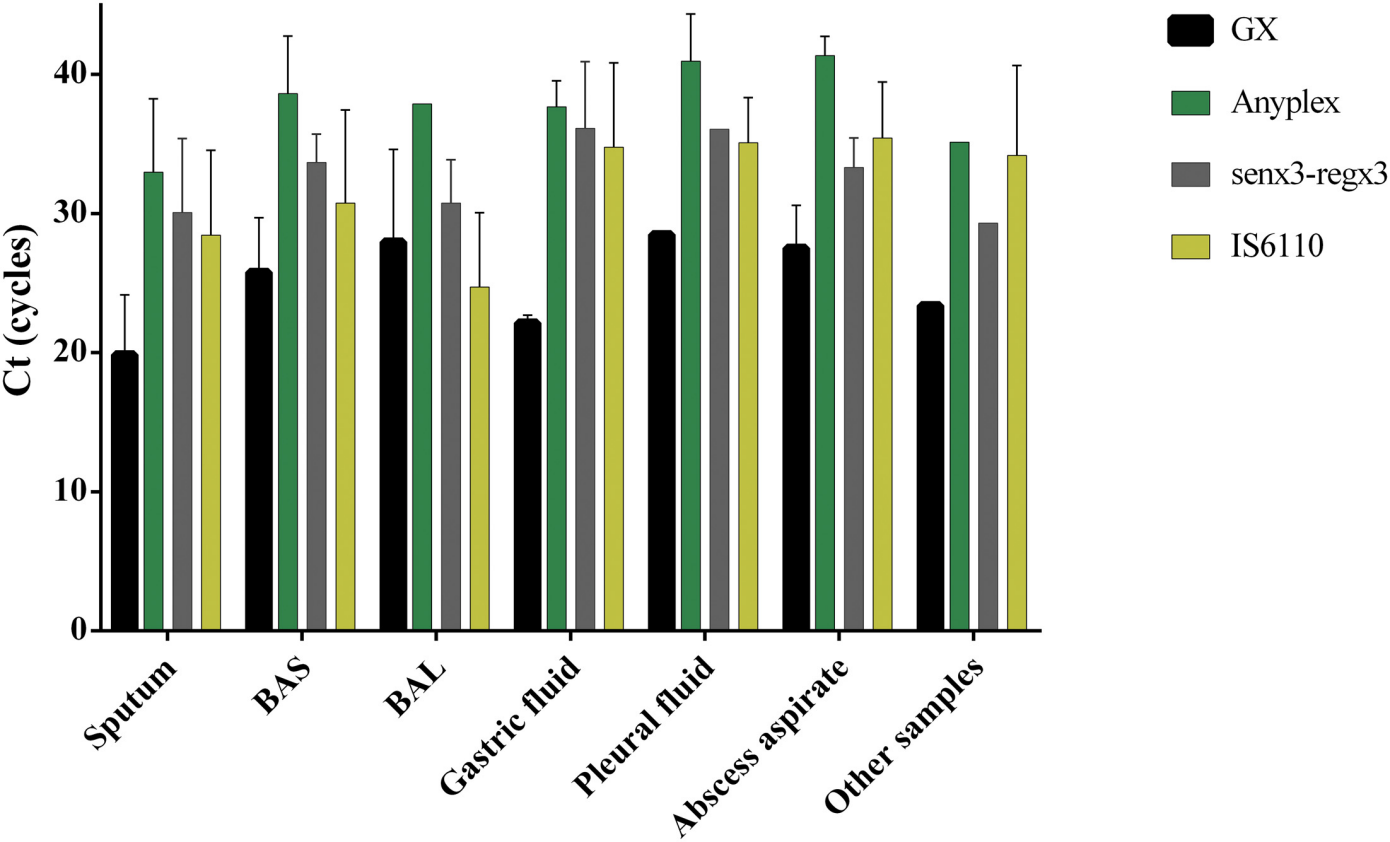
The mean values of Ct of positive TB samples by different assays. Other samples included CSF, lymph node and urine.

The mean Ct values for the four strategies studied varied according to the type of sample analysed. Except in the case of BAS samples, the earliest detection always corresponded to GX and the latest to Anyplex, with differences in the Ct ranging from 9.76 to 13.84 cycles The difference in the Ct between the senX3-regX3 and IS6110 assays with respect to GX was 2.79–14.00 and 3.2–12.64 cycles, respectively (Fig 3).

Finally, the total processing time to generate a result was around 2.5 hours for GX, 4.5 hours for senX3-regX3 and IS6110, and 5 hours for Anyplex.

### DNA concentrations and purity of clinical samples

**Fig 4:**
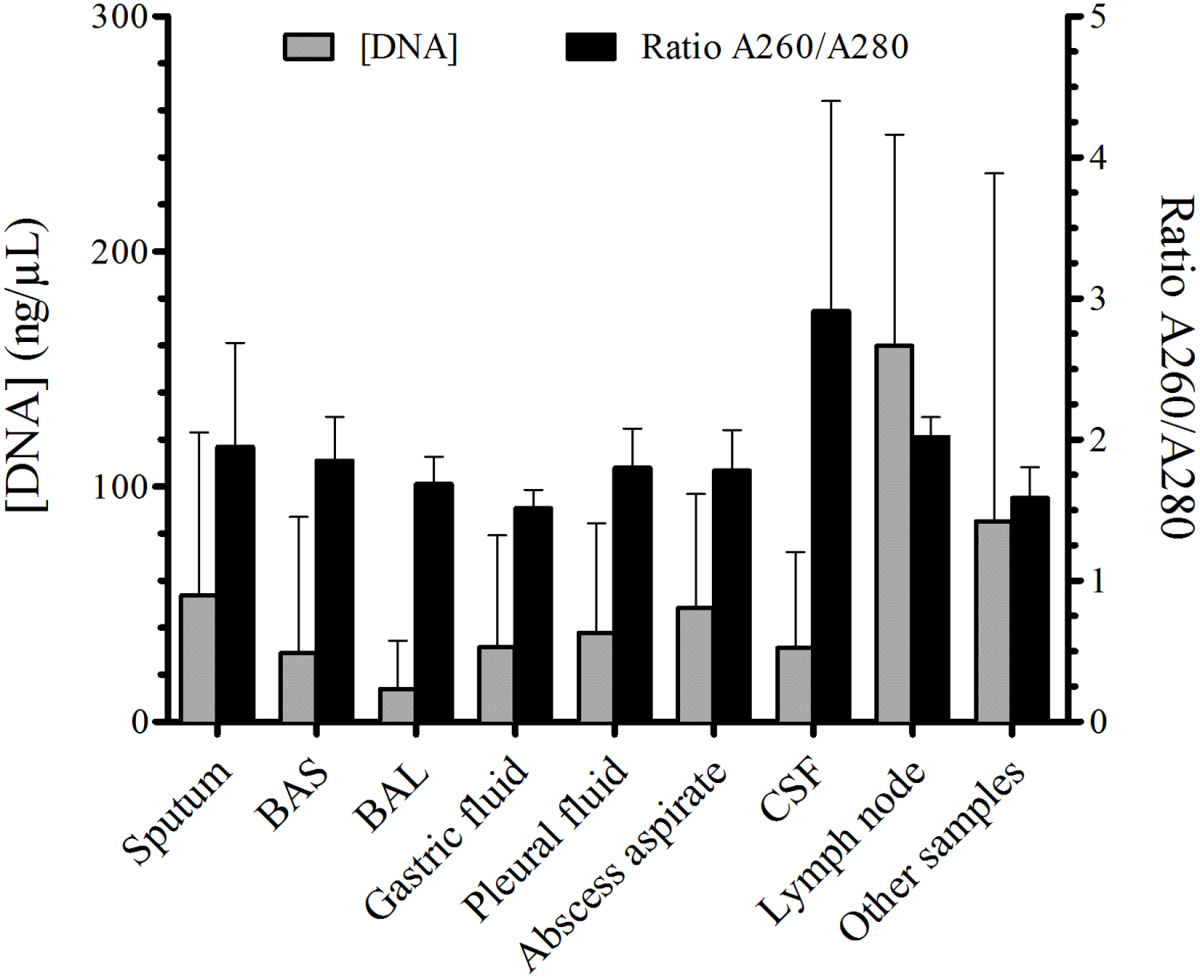
The DNA concentrations and purity of different clinical samples. Other samples included CSF, lymph node and urine.

All the sample types had a good purity, ranging between 1.7 and 2.0, except the CSF samples which had a purity of 2.91 ± 1.49. Excluding one case of osteoarticular TB with fine needle aspiration, in which the amount of DNA was low, 0.66 ng/μl, the mean amount of DNA was suitable, ranging from 17.64 to 53.83 ng/μl. The amount and purity of DNA depending on the sample type is shown in Fig 4.

## Discussion

Nucleic acid amplification tests (NAATs) are increasingly used for the diagnosis of TB in clinical laboratories. Many countries and institutions now recommend NAATs as standard practice, and several of these tests are now available for the diagnosis of TB, both commercial and in-house. However, the former are often too expensive for low-income countries and the latter are not yet sufficiently standardized to predict the results consistently and reliably. Moreover, the diagnostic yield of the most commonly used NAATs in clinical practice, varies notably depending on the sample, its processing, the smear status and the processing laboratory. Accordingly, the objective of this study was to evaluate the efficiency and effectiveness of three different strategies; molecular beacons with GX, READ technologies with Anyplex, and SYBR Green I based in-house senX3-regX3and IS6110 real time PCR, for the detection of MTBC in a representative sample of patients with TB in a clinical laboratory setting.

The LOD of the Anyplex, IS6110 and senX3-regX3 assays was approximately 2 GE, suggesting that these three assays could detect a very low bacterial load in clinical samples. The LOD of GX, with the method used in our study, was 2.13E+03 CFU/ml, although a greater range of dilutions could be performed to establish the lower limit, between 2.13E+03 and 2.13E+02 CFU/ml. Our results are consistent with those of Miller et al. [19], who established the LOD at 876 CFU/ml with the *M*. *tuberculosis* strain. Equally, Dharan et al. [20] obtained a LOD of 832 CFU/ml with *M*. *tuberculosis* in a sputum model, with the same proportion of sample reagent used as in this work. In contrast, Helb et al. [21] reported a LOD of 131 CFU/ml with sputum samples spiked with *M*. *tuberculosis* cells.

The small differences in Tm found with IS6110 and senX3-regX3 depending on the LC instrument used were attributable, in our opinion, to the different concentrations of the components of each mastermix and the temperatures cheduled [22–23].

The overall diagnostic yield of the assays was better for senX3-regX3and GX than for IS6110 and Anyplex. The sensitivity of IS6110 was substantially higher than the sensitivity of the other strategies used, ranging from 89.7–99.8%. However, the poor specificity of IS6110 affected its overall performance. This lack of specificity of the IS6110 target, one of the most widely used for the detection of MTBC, has already been described [19, 24–25]. In fact, Sankar et al. [26] compared *in silico* the available nucleotide sequences of IS6110 of *M*. *tuberculosis* strains and observed variations between strains and even within copies of the same strain. Thus, the sensitivity and specificity of the target depend on the region chosen for primer design and false-positive results can be explained by homology with other species of *Mycobacterium* and/or other microorganisms with certain DNA regions conserved during evolution. These data are consistent with the findings of the present study and could explain the problems of specificity found with IS6110 and Anyplex, especially in pulmonary samples where the prevalence of NTM colonization is high.

In the smear-positive samples, both the pulmonary and the extrapulmonary samples, the sensitivity of the four PCR assays was excellent, but it decrease significantly in smear-negative samples. This is constant in all NAAT studies of the diagnosis of TB [27–29]. Thus, for pulmonary and extrapulmonary smear-positive samples the sensitivity of the GX and senX3-regX3 assays was 100%. Conversely, for GX and senX3-regX3, respectively, the sensitivity was 53.3% and 76.7% in pulmonary smear-negative samples and 66.7% and 55.6% in extrapulmonary smear-negative samples.

Concerning GX, the results of this study were in fact very similar to those reported recently by Steingart et al.[28] in a large meta-analysis, where the pooled sensitivity for pulmonary smear-positive and smear-negative samples was 98% and 67% respectively [28]. Likewise, Maynard-Smith et al. [29], in a systematic review of the diagnostic efficacy of GX in pulmonary and extrapulmonary samples, reported sensitivity values of around 95% for smear-positive samples and 69% for smear-negative samples. As far as we are aware though, only two studies have analysed the efficacy of the senX3-regX3 target in the diagnosis of TB. In a study including 54 samples from patients with confirmed TB and 61 control samples, Broccolo et al. reported a sensitivity of 94% and a specificity of 100%, using an in-house senX3-regX3 Taqman assay [24]. Likewise, Lee et al. analysed 129 paraffin-embedded human tissue specimens, 63 TB and 66 control cases, and reported a sensitivity of 74.6% and a specificity of 98.5% [30]. Thus, the results obtained in our study are consistent with those reported in the literature and the differences found between the GX and senX3-regX3 assays seem to be related to the efficiency of the amplification of each target as well as the DNA extraction method and the initial sample volume. Santos et al. [31] showed that the DNA extraction method affects the molecular diagnostic yield of pleural TB. These authors compared nine methods of extraction in a model system of TB by RT-PCR analysis but were only able to detect *M*. *tuberculosis* in all the pleural samples in two methods: the manual extraction columns (Qiagen) and automatic TNAI (Roche). Likewise, Aldous et al. [32] demonstrated that the extraction methods used in sputum samples influence the performance of real time PCR techniques.

The amount and purity of the DNA extracted from the different clinical samples processed suggests that the extraction method used in our study was adequate for all the samples assayed, except for CSF. Nevertheless, the lower sensitivity of all NAATs in clinical samples taken from sterile cavities is well known, possibly related to the presence of inhibitors and the low TB bacterial load in these sites [31, 33–34].

The results of this study should be interpreted bearing in mind its limitations. First, although the number of patients included in the study was not very large, it was nonetheless no smaller than many of the studies included in a recent meta-analysis about GX published by the Cochrane Collaboration [28]. Second, as a consequence of the sample size the number of patients with extrapulmonary TB was also limited, though the percentage of these with respect to the total number of patients with TB was 13.2%, a figure that is within the usual range for large series of extrapulmonary TB, which varies from 10–42% of the total number of cases of TB [29]. In addition, the sites of extrapulmonary TB in the patients included in our study were very representative of the situation in usual clinical practice [35]. On the other hand, this study has the strength of a prospective design and the consecutive inclusion of all patients diagnosed with TB in our hospital over the 18-month recruitment period. This impedes any kind of selection bias and possible problems inherent to the prolonged conservation of the samples prior to their processing. Finally, both the Anyplex and the GX assays were performed in our laboratory as part of daily practice, which, to a certain extent, increases the external validity of our results.

In conclusion, our results suggest that real-time PCR assays targeting IS6110 lack the desired specificity, which is probably related to the choice of the amplified region. The Xpert MTB/RIF and in-house senX3-regX3 assays are sensitive and specific for the detection of MTBC in both pulmonary and extrapulmonary samples of TB. The senX3-regX3 assay could be used as a complementary tool in doubtful cases with smear-negative results.

## Supporting Information

S1 Fig The standard curves of the senX3-regX3 (A) and IS6110 (B) assays using different instruments. (TIF)

S2 Fig The mean values of Tm (°C) of the senX3-regX3 (X3) and IS6110 (IS) assays using different instruments and samples. RS, reference strains; CS, clinical samples.
(TIF)
