# Supplementary figures and images for: Comparative Study of a Real-Time PCR Assay Targeting senX3-regX3 versus Other Molecular Strategies Commonly Used in the Diagnosis of Tuberculosis
Source: PLoS One. 2015 Nov 24;10(11):e0143025. doi: 10.1371/journal.pone.0143025 (PMC4658205; doi:10.1371/journal.pone.0143025)

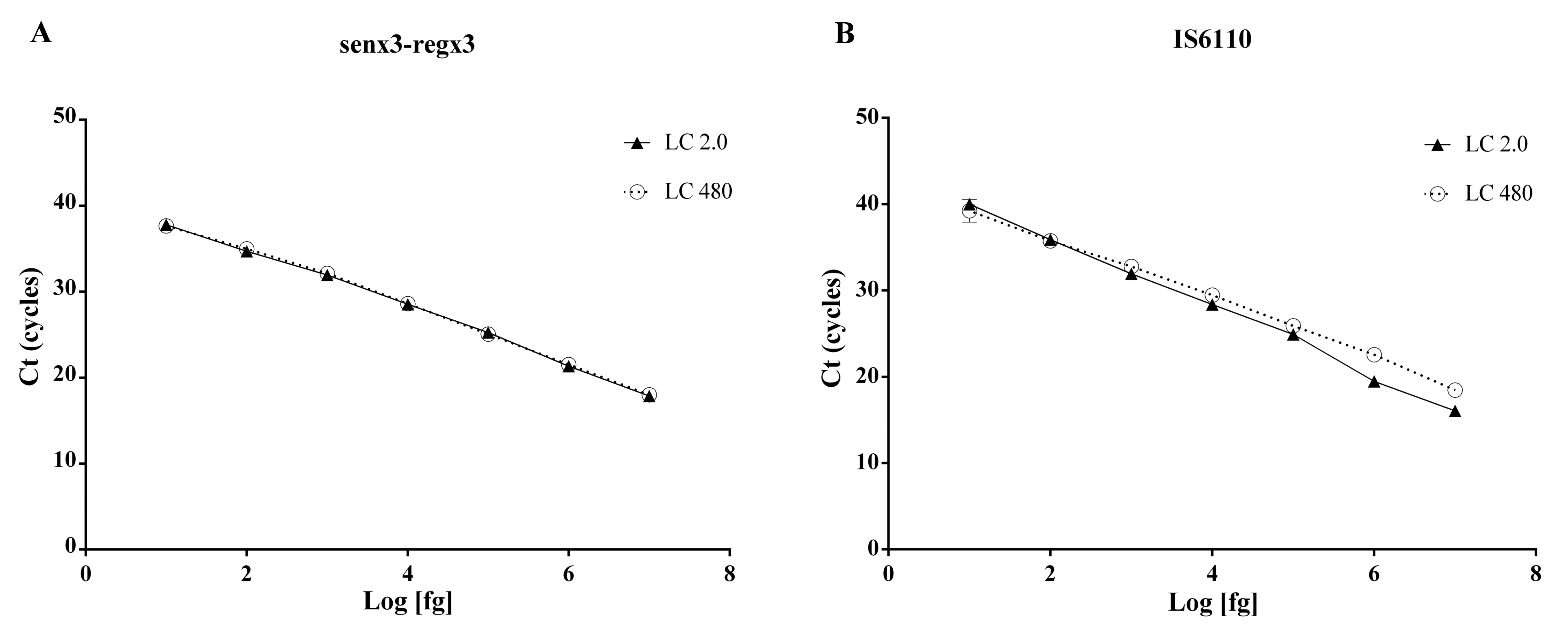

Supplement: S1 Fig — (TIF) [file pone.0143025.s001.tif]

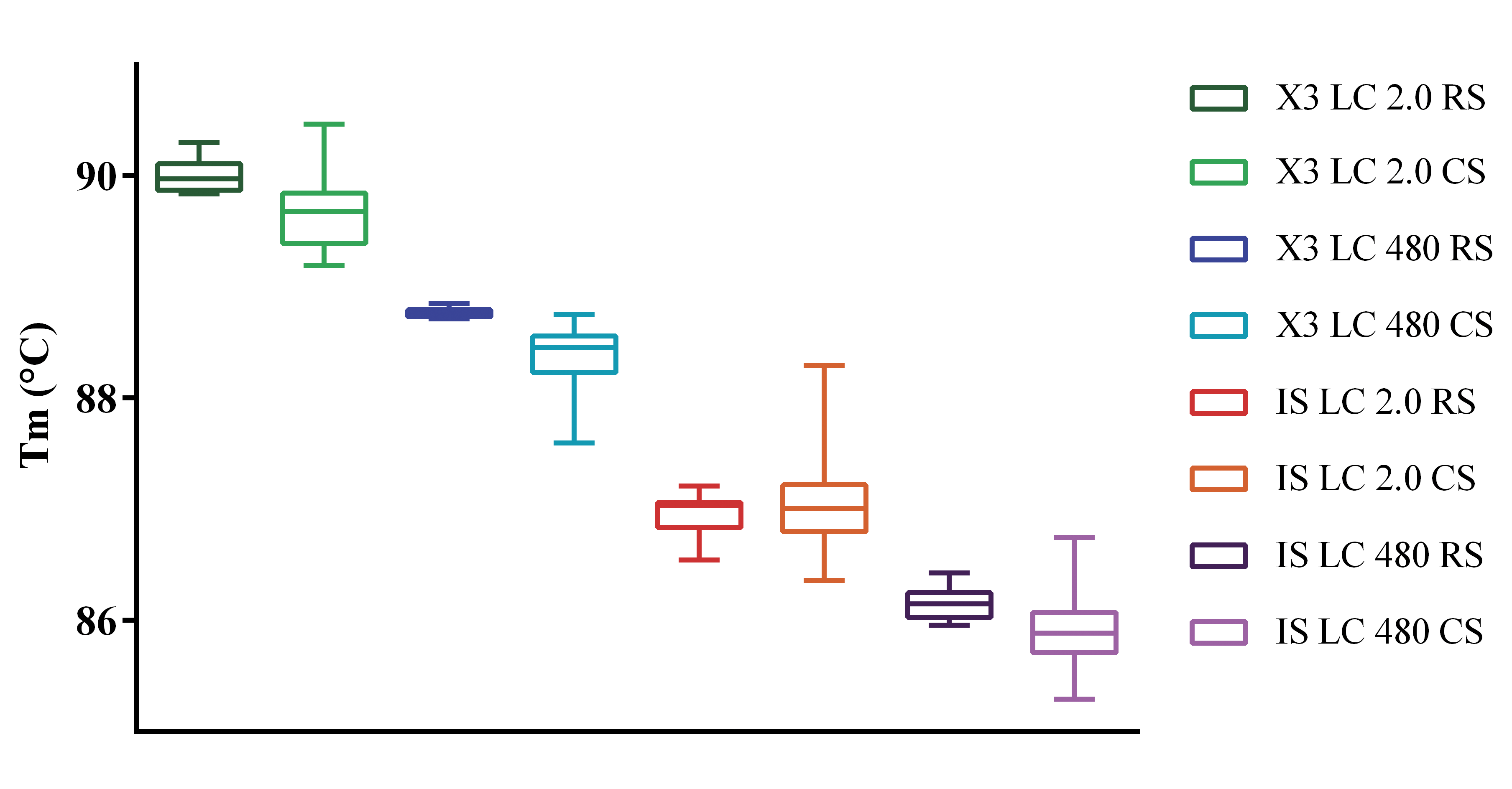

Supplement: S2 Fig — RS, reference strains; CS, clinical samples. (TIF) [file pone.0143025.s002.tif]
